# Overexpression of Cotton *GhMPK11* Decreases Disease Resistance through the Gibberellin Signaling Pathway in Transgenic *Nicotiana benthamiana*

**DOI:** 10.3389/fpls.2016.00689

**Published:** 2016-05-23

**Authors:** Fang Wang, Chen Wang, Yan Yan, Haihong Jia, Xingqi Guo

**Affiliations:** State Key Laboratory of Crop Biology, College of Life Sciences, Shandong Agricultural UniversityTai’an, China

**Keywords:** *Gossypium hirsutum* L., *GhMPK11*, disease resistance, GA_3_, ROS

## Abstract

Many changes in development, growth, hormone activity and environmental stimuli responses are mediated by mitogen-activated protein kinase (MAPK) cascades. However, in plants, studies on MAPKs have mainly focused on *MPK3*, *MPK4* and *MPK6*. Here, a novel group B MAPK gene, *GhMPK11*, was isolated from cotton (*Gossypium hirsutum* L.) and characterized. Both promoter and expression pattern analyses revealed that *GhMPK11* is involved in defense responses and signaling pathways. *GhMPK11* overexpression in *Nicotiana benthamiana* plants could increase gibberellin 3 (GA_3_) content through the regulation of GA-related genes. Interestingly, either *GhMPK11* overexpression or exogenous GA_3_ treatment in *N. benthamiana* plants could enhance the susceptibility of these plants to the infectious pathogens *Ralstonia solanacearum* and *Rhizoctonia solani*. Moreover, reactive oxygen species (ROS) accumulation was increased after pathogen infiltration due to the increased expression of ROS-related gene respiratory burst oxidative homologs (*RbohB*) and the decreased expression or activity of ROS detoxification enzymes regulated by GA_3_, such as superoxide dismutases (SODs), peroxidases (PODs), catalase (CAT) and glutathione *S*-transferase (GST). Taken together, these results suggest that *GhMPK11* overexpression could enhance the susceptibility of tobacco to pathogen infection through the GA_3_ signaling pathway via down-regulation of ROS detoxification enzymes.

## Introduction

In contrast with animals, when confronted with various environmental challenges, plants cannot escape danger because they are sessile. To adapt to different stresses, plants have evolved sophisticated signaling pathways to transduce biotic or abiotic stimuli into proper cellular responses (Xie et al., 2014). Among those pathways, mitogen-activated protein kinase (MAPK) cascades that are conserved in all eukaryotes function as a mechanism to sense invading pathogens and then transduce internal or external signals to downstream effectors, such as transcription factors.

Typical MAPK cascades contain MAPK kinase kinases (MAPKKKs), MAPK kinases (MAPKKs) and MAPKs. MAPKKKs can activate MAPKKs by phosphorylating a dual-specificity Ser/Thr or Tyr residue (Jonak et al., 2002; Rodriguez et al., 2010). Then, activated MAPKKs can phosphorylate Thr and Tyr residues in the activation loop (A-loop) of MAPKs (Xu et al., 2014). Finally, the activated MAPKs interact with downstream transcription factors and transduce signals into cellular responses typically by altering gene expression (Widmann et al., 1999; Ichimura et al., 2002). In *Arabidopsis*, 20 MAPKs have been identified. Based on their sequence homology and the conserved phosphorylation motifs, MAPKs can be divided into four groups: A, B, C, and D (MAPK Group, 2002). Group A-C MAPKs contain a TEY motif in their conserved domains, while group D MAPKs contain a TDY motif (Rodriguez et al., 2010; Meng and Zhang, 2013). MAPKs in groups A and B also have a conserved common docking (CD) domain (Tanoue et al., 2000).

Plant MAPK cascades are induced by numerous environmental stresses, such as wounding, drought, salinity, cold and diverse pathogens (Pitzschke et al., 2009; Rodriguez et al., 2010; Sinha et al., 2011; Rasmussen et al., 2012; Šamajová et al., 2013). Group A MAPKs have been found to be involved in environmental and hormonal responses. *GhMPK6a* (*Gossypium hirsutum*) can reduce tolerance to osmotic stress and bacterial infection and plays an important role in development (Li et al., 2013). In *Arabidopsis*, *AtMPK3* and *AtMPK6* participate in pathogen resistance and abiotic stress (Rodriguez et al., 2010; Meng and Zhang, 2013). Of the group C MAPKs, the expression level of *PsMPK2* (*Pisum sativum* L.) is induced by wounding, JA, abscisic acid (ABA), and H_2_O_2_ (Ortiz-Masia et al., 2008). *AtMPK1* and *AtMPK2* have been reported to be involved in pathogen signaling (Dóczi et al., 2007). In addition, *GhMPK7* might participate in SA-regulated broad-spectrum resistance to pathogen infection (Shi et al., 2010). *AtMPK9*, a group D MAPK, is involved in ABA signaling through ROS homeostasis and calcium and anion channels (Jammes et al., 2009). A recent study revealed that *GhMPK17* might be involved in plant responses to high salinity, osmotic stresses and ABA signaling (Zhang et al., 2014). *AtMPK4*, a group B MAPK, regulates the levels of plant hormones and negatively regulates systemic acquired resistance and innate immunity in plants (Petersen et al., 2000; Brodersen et al., 2006; Brader et al., 2007; Gao et al., 2008). However, studies on other group B members are limited.

Mitogen-activated protein kinases exert their functions in association with diverse phytohormones. SA, JA and ET are well-established phytohormones involved in disease responses (Yang D.L. et al., 2008). Development-related hormones such as brassinosteroid (Nakashita et al., 2003), cytokinin (Siemens et al., 2006), auxin (Zhang et al., 2007), and ABA (Adie et al., 2007) are associated with disease resistance. The functions of these hormones in response to diseases vary, suggesting that plants might use diverse signaling pathways to manage defense responses during pathogen infection. GA signaling also functions as a stress signal similar to other phytohormones, such as ABA or SA, under adverse conditions (Achard et al., 2006; Willige et al., 2011; Gallego-Bartolomé et al., 2012; Golldack et al., 2013; Colebrook et al., 2014). However, previous studies have mainly focused on GA’s function in plant resistance to drought, salinity and other abiotic stresses, and its function in disease responses has been neglected (Achard et al., 2006; Shan et al., 2007; Li et al., 2012).

Cotton (*G. hirsutum*) is one of the most important economic crops and serves as an important source of food, fiber, oil and biofuel (Zhang et al., 2002; Sunilkumar et al., 2006; Chen et al., 2007). Furthermore, *G. hirsutum* produces more than 95% of the annual cotton crop worldwide, and its research value for genome size evolution studies cannot be ignored (Grover et al., 2004). In this study, a novel group B MAPK gene, *GhMPK11* from *G. hirsutum*, was isolated and characterized. The transcription level of *GhMPK11* could be induced by various stresses, and overexpression of *GhMPK11* led to a higher level of GA_3_ than that in control plants. Furthermore, *GhMPK11* overexpression and exogenous GA_3_ treatment could reduce plants resistance to pathogens. All of these results suggest that *GhMPK11* may increase plants susceptibility to pathogens due to a higher level of GA_3_ *in vivo*. This study increases our understanding of MAPK signaling in cotton and indicates that GA signaling may have a role in disease responses.

## Materials and Methods

### Plant Materials and Treatments

Cotton (*G. hirsutum* L. cv lumian 22) seeds were grown in an incubator at 25 ± 1°C with a 16 h light/8 h dark cycle (light intensity of 200 μmol m^-2^ s^-1^; relative humidity of 60–75%). The 7-day-old cotton seedlings were sprayed with 10 mM H_2_O_2_, 100 μM ABA and 100 μM GA_3_. For pathogen treatment, 7-day-old cotton seedlings were inoculated with bacterial suspensions of *Ralstonia solanacearum* (OD_600_ = 0.6–0.8) and conidial suspensions of *Rhizoctonia solani* (10^5^ conidia/mL) using the root dip method. The treated cotyledons were harvested at the appropriate times as indicated, frozen in liquid nitrogen and stored at -70°C for RNA extraction. Each treatment was repeated at least twice. *N. benthamiana* seeds were surface-sterilized, planted in soil, and maintained under a 16 h light/8 h dark photoperiod at 25°C. Three to four-leaf-stage *N. benthamiana* seedlings were transplanted into pots with soil and maintained under glasshouse conditions.

### Cloning of the Full Length cDNA, Genomic Sequences and 5′ Flanking Region of *GhMPK11*

The cDNA and genomic DNA were isolated as described previously (Yu et al., 2012). Total RNA was extracted from the leaves of cotton seedlings using the CTAB method (Hu and Yu, 2007). To obtain the internal conserved fragment of *GhMPK11*, primers MF and MR were designed based on the nucleotide sequences and amino acids that are conserved among TcMPK4 (*Theobroma cacao*), AtMPK11 and AtMPK4 (*Arabidopsis thaliana*) and NaMPK4 (*Nicotiana attenuate*). Then, RT-PCR was performed to clone an internal fragment of *GhMPK11*. Next, primers 5N, 5W, 3N and 3W were designed based on the *GhMPK11* fragment, and then TAIL PCR was performed to amplify the 5′ flanking region according to Liu and Chen (2007). The primers used in this study are shown in **Supplementary Table S1**. All products were purified, cloned into the pEASY-T3 vector (TransGen Biotech, China), transformed into the *Escherichia coli* strain DH5α and then sequenced. All sequencing was performed by BioSune using an ABI 3730 XL sequencer.

### RNA Extraction

Total RNA was extracted from cotton seedlings according to the CTAB method described by Hu and Yu (2007). Total RNA was extracted from *N. benthamiana* seedlings using TRIzol Reagent (Invitrogen, Carlsbad, CA, USA) and digested with RNase-free DNaseI (Promega, Madison, WI, USA). The first strand cDNA was synthesized using the EasyScript First-Strand cDNA Synthesis SuperMix (TransGen Biotech, Beijing, China).

### Expression Pattern of *GhMPK11* Using Quantitative PCR

Total RNA was extracted from all experimental samples, and the first strand cDNA was synthesized as described above. Quantitative PCR (qRT-PCR) was performed as previously described (Wang et al., 2014). All primers used are listed in **Supplementary Table S1**. *G. hirsutum* polyubiquitin (*UBI*) or *N. benthamiana* β*-actin* genes were used as the standard control. The relative expression level of *GhMPK11* was analyzed using the comparative CT method (2^-ΔΔCT^ method), and three replicates of each sample were analyzed. Prism 5 software (GraphPad Software, Inc.) was used to determine significant differences.

### Subcellular Localization of GhMPK11

The ORF of GhMPK11 was inserted upstream of the N-terminus of the GFP gene following the CaMV35S promoter. The recombinant vector was transformed into onion epidermal cells using the particle bombardment method as described by Yu et al. (2012). Then, the tissues were incubated in the dark at 25°C for 12 h. Nuclei were stained with 100 μg/mL 4′,6-diamidino-2-phenylindole (DAPI) (Solarbio, Beijing, China) in phosphate-buffered saline for 10 min. The 35S::GFP construct was used as a control. In addition, the two recombinant plasmids were transferred into *Agrobacterium tumefaciens* strain GV3101. *Agrobacterium* cells were harvested by centrifugation, resuspended in infiltration buffer (10 mM MES, pH 5.7, 10 mM MgCl_2_, and 150 mM acetosyringone) and adjusted to a final OD_600_ of 1.0. After the *Agrobacterium* mixture was incubated for 3 h at room temperature in the dark, it was infiltrated into the leaves of 6-week-old *N. benthamiana* plants with a syringe. The 35S-GFP plasmid was used as a control. Fluorescence was observed 3 to 4 days after infiltration using a confocal laser scanning microscope (LSM 510 META, ZEISS, Germany).

### Vector Construction and Genetic Transformation

The vector construction and genetic transformation were performed as previously described by Yu et al. (2012). Plants transformed with pBI121-GFP were used as controls. Transformation of the tobacco plants was confirmed by PCR. The transgenic T_2_ lines and vector plants were used for further experiments.

### GA Content Measurement

Leaves were detached from plants OE *GhMPK11* or the control were grounded in liquid nitrogen, and then soaked in 95% chromatographic methanol at 4°C overnight. The solution was filtered through a 0.45 μm membrane, and the GA_3_ content was measured via Agilent 1200 rapid resolution liquid chromatography (Agilent Technologies, Waldbronn, Germany). The mobile phase was 0.4 % (v/v) acetic acid + chromatographic methanol. Chromatography was performed at 254 nm at 30°C with a flow rate of 1.0 cm^3^ min^-1^. The marker for GA_3_ was accurately weighed and dissolved in chromatographic methanol to provide serial concentrations ranging from 0.0133 to 13.3 mg/mL. The standard curve was analyzed using the peak area, and the GA_3_ content was calculated.

### DAB and NBT Staining Assays

For DAB staining, leaves were soaked in DAB solution (1 mg/mL, pH 3.8) in the dark at 25°C for 12 h. Then, the leaves were incubated in 95% ethanol overnight to remove the chlorophyll. For NBT staining, the leaves were soaked in NBT solution (0.1 mg/mL) in the dark for 12 h at 25°C. Then, the leaves were incubated in 95% ethanol overnight to remove the chlorophyll. Seedlings treated with water were used as controls.

### GUS Histochemical Staining Assay

Transgenic *Arabidopsis* plants harboring the *ProGhMPK11::GUS* construct were generated using the floral dip method. T_2_ progeny were used for promoter activity analyses and stained with the GUS histochemical staining buffers as previously described (Jefferson, 1987).

### Disease Resistance of the Transgenic Plants

For bacterial infection, the detached leaves of 8-week-old seedlings were inoculated with suspensions of *R. solanacearum*, a Gram-negative plant pathogenic bacterium (OD_600_ = 0.6–0.8). The bacteria were cultured in Luria–Bertani (LB) broth overnight at 37°C, harvested by centrifugation, and resuspended in sterile tap water. For fungal infection, *R. solani* was cultured on potato dextrose agar (PDA) medium for 2 weeks at 28°C, and the spores were then suspended in sterile tap water. *R. solani* spore suspensions (10^5^ spores/mL) were infiltrated into leaves detached from 8-week-old T_2_ transgenic and control seedlings using a needleless syringe. At least three independent experiments were performed for each pathogen.

### Pathogen Growth Assays

Total RNA was extracted from each sample using TRIzol Reagent (Invitrogen, Carlsbad, CA, USA) according to the manufacturer’s instructions. The abundance of the fungus (*R. solani*) was estimated by the ITS gene copy number through qPCR using the primer pair ITS1/ITS4 (White et al., 1990). The bacterial (*R. solanacearum*) abundance was estimated by the 16S rRNA gene copy number through qPCR using the primer pair Eub338 and Eub518 (Rasche et al., 2011). Amplification reactions were carried out with SYBR Premix Ex Taq (TaKaRa, Japan) in a total volume of 20 μL. Standard curves were obtained using serial dilutions of a known copy number of plasmids containing an ITS or 16S rRNA gene fragment, and these curves were linear from 9.77 × 10^3^ to 9.77 × 10^8^ gene copies/μL (*R*^2^ = 0.998; ITS gene) and gene copies/μL (R^2^ = 0.998; 16S rRNA gene). All samples were analyzed in triplicate.

### Enzyme Activity Assays and Oxidative Stress Experiments

For the enzyme activity assays, the leaves of transgenic and control plants were inoculated with *R. solanacearum* or *R. solani* and then tested for SOD, POD and CAT activity as previously described (Yang L. et al., 2008). Oxidative stress experiments were performed according to Lu et al. (2013) with a modification of the MV concentration.

## Results

### Isolation and Sequence Analysis of *GhMPK11*

**FIGURE 1:**
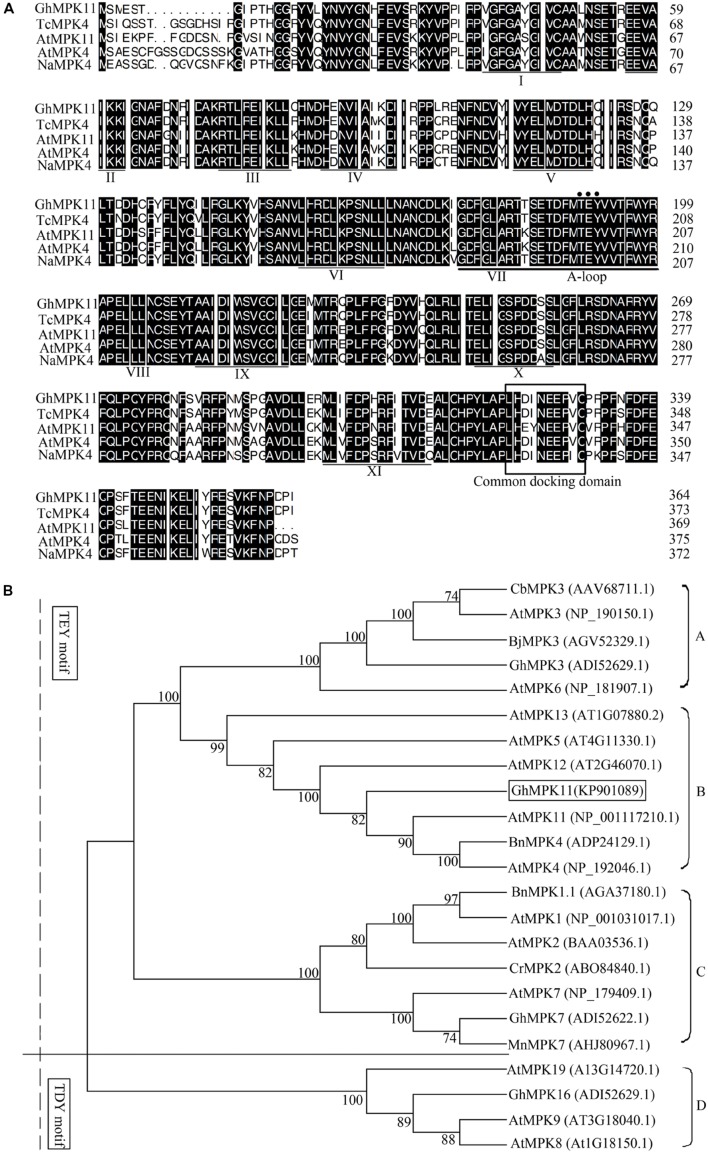
**Sequence and phylogenetic analyses of GhMPK11 (GenBank accession no. KP901089).** **(A)** Multiple alignments of the GhMPK11 protein with TcMPK4 (EOX95887.1), AtMPK11 (NP_001117210.1), AtMPK4 (NP_192046.1) and NaMPK11 (ADT91692.1). Identical amino acids are shaded in black. The conserved subdomains are indicated by numerals (I–XI) at the bottom of the sequences. The activation loop (A-loop) is underlined and contains the phosphorylation motif (TEY motif) marked by a circle. The common docking domain, which is the binding site of MAPKKs and GhMPK11, is boxed. **(B)** Phylogenetic analysis of MAPK proteins from different species. GhMPK11 is boxed. The GenBank accession numbers are indicated in parentheses. The GenBank IDs follow the protein names.

The full-length cDNA of *GhMPK11* (KP901089) contained a 95-bp 5′ untranslated region (UTR), 40-bp 3′ UTR and 1098-bp ORF that encoded a 365-amino-acid protein. Multiple alignments indicated that GhMPK11 possesses a conserved TEY motif in the activation loop, 11 conserved subdomains, and a CD domain (**Figure 1A**). Multiple alignments also demonstrated high identities (84.18–92.78%) with homologous sequences, including TcMPK4 from *T. cacao*, AtMPK11 and AtMPK4 from *A. thaliana* and NaMPK4 from *N. attenuata*. To investigate the evolutionary relationships among MAPK proteins from different species, a phylogenetic analysis based on the amino acid sequences was performed using the neighbor-joining method and MEGA software version 4.1. The results demonstrated that *GhMPK11* belongs to the group B MAPK family (**Figure 1B**).

### Subcellular Localization of GhMPK11

**FIGURE 2:**
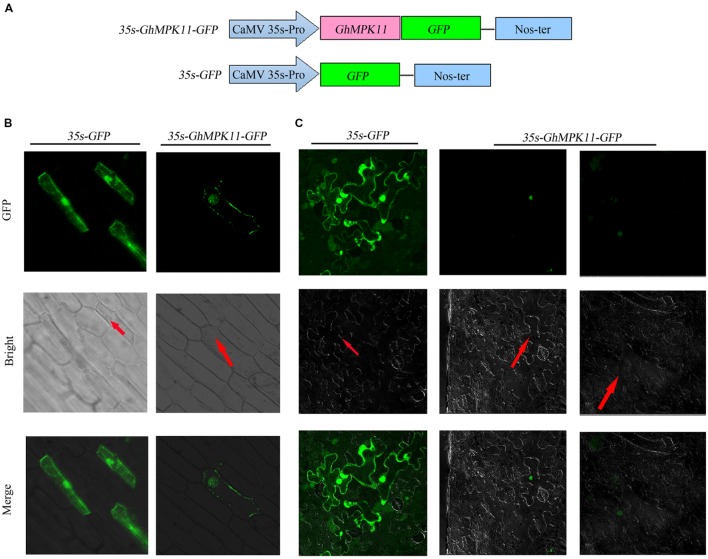
**The subcellular localization of GhMPK11 protein transiently expressed in onion epidermal cells and tobacco cells.** **(A)** Schematic diagram of the *35S-GhMPK11-GFP* fusion construct and the control *35S-GFP* construct. GFP was fused to the C terminus of *GhMPK11*. Transient expression of the *35S-GhMPK11-GFP* and *35S-GFP* constructs in onion epidermal cells **(B)** and tobacco cells **(C)**. Red arrows indicate the location of the nucleus. Green fluorescence was observed using a confocal microscope. Bar = 200 mm.

The Plant-mPLoc program was used to predict the subcelluar localization of GhMPK11 and indicated that GhMPK11 localizes in the nucleus. However, CELL version 2 predicted that GhMPK11 primarily localizes in the cytoplasm. To investigate the localization of GhMPK11, a biolistic transformation system was used for a transient assay. Two constructs, 35S-GhMPK11::GFP and 35S-GFP (**Figure 2A**), were introduced individually into onion epidermal cells. As shown in **Figure 2B**, fluorescent signals were found in the nucleus, and DAPI staining was also detectable in the nucleus (**Supplementary Figure S1**). Additionally, the 35S-GhMPK11::GFP and 35S-GFP constructs were transformed individually into tobacco cells, and fluorescence signals were found in the nucleus (**Figure 2C**). These results suggested that GhMPK11 localizes in the nucleus.

### Expression Patterns of *GhMPK11*

**FIGURE 3:**
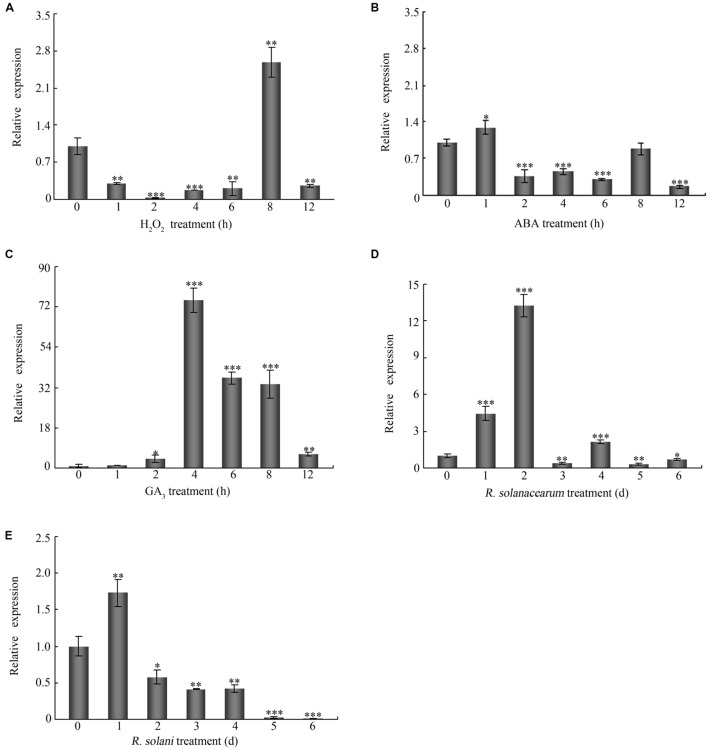
**The expression patterns of *GhMPK11*.** Seven-day-old cotton seedlings obtained from hydroponic culture were treated with 10 mM H_2_O_2_ **(A)**, 100 μM ABA **(B)**, 100 μM GA_3_ **(C)**, *R. solanacearum* **(D)** and *R. solani* **(E)**. The *G. hirsutum* polyubiquitin (UBI) gene was employed as an internal control. The data are the means ± SD from three independent experiments. The asterisks indicate statistically significant differences according to Student’s *t*-test (^∗^*P* < 0.05; ^∗∗^*P* < 0.01; ^∗∗∗^*P* < 0.001).

To study the effects of signaling molecules and biotic stresses on the expression of *GhMPK11*, 7-day-old cotton seedlings were treated with H_2_O_2_, ABA, GA_3_, *R. solanacearum* and *R. solani*. *GhMPK11* expression increased strongly at 8 h under H_2_O_2_ treatment (**Figure 3A**). ABA treatment led to a slight increase in *GhMPK11* transcription levels at 1 h after treatment (**Figure 3B**), while GA_3_ treatment sharply enhanced the expression level of *GhMPK11* at 4 h after treatment (**Figure 3C**). Following *R. solanacearum* treatment, the expression level of *GhMPK11* was rapidly induced within 2 h (**Figure 3D**). *R. solani* treatment also induced the transcription of *GhMPK11* at 1 h, and then a gradual reduction was observed (**Figure 3E**). These results indicated that *GhMPK11* could be induced by pathogens and signaling molecules.

### Promoter Analysis of *GhMPK11*

To characterize the underlying mechanism by which *GhMPK11* responds to multiple stresses, a 1309-bp fragment was cloned upstream of the transcription start site (KP901088). The PlantCARE database was used to analyze the *cis*-acting regulatory elements. Some putative *cis*-acting regulatory elements, such as the defense-responsive elements WRKY71OS and TC-rich repeats, MBSI and the GA-response element GAREAT TC-rich repeats were found in this region. Some identified *cis*-elements are listed in **Table 1**.

**Table 1:** Putative *cis*-acting regulatory elements in the *GhMPK11* promoter.

	*cis*-Element	Position	Sequence (5′-3′)
Stress response elements	HSE	-1299(-)	AAAAAATTTC


	LTR	-792(-)	CCGAAA
	MBSI	-196(-)	aaaAaaC(G/C)GTTA
	MBS	-190(-), -995(+)	C/TAACTG
	TC-rich repeats	-1021(-)	ATTCTCTAAC
Hormones	GAREAT	-376(+), -1184(-)	TAACAAR
	TATCCAOSAMY	-95(+)	TATCCA
	WRKY71OS	-401(+), -1093(+), -1212(+), 1206(+)	TGAC/GTCA
Sugar	WBOXHVISO1	-401(+), -1093(+), -1212(+)	TGACT
	SREATMSD	-94(+)	TTATCC
Light regulation elements	3-AF1 binding site	-829(+)	AAGAGATATTT


	ACE	-196(-), -674(+)	AAAACGTTTA
	AE-box	-801(-)	AGAAACTT
	Box4	-911(+), -1153(+)	ATTAAT
Development-related elements	EIRE	-950(+)	TTCGACC


	CAAT-motif	-975(+), -896(+), -599(+), -890(+)	CAAT
	Skn-1-motif	-871(-), -1032(+)	GTCAT


**FIGURE 4:**
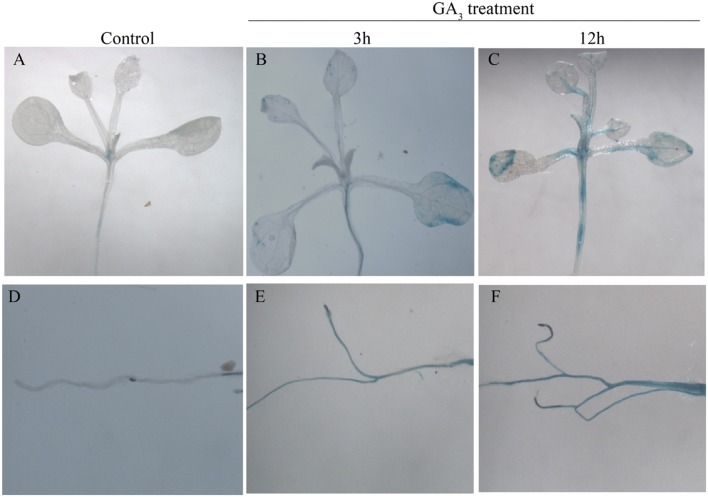
**β-glucuronidase activity analysis in *ProGhMPK11::GUS* plants in response to GA_3_.** **(A,D)** No treatment. **(B,E)** Treated with GA_3_ for 3 h. **(C,F)** Treated with GA_3_ for 12 h.

To analyze the promoter activity of *GhMPK11*, *ProGhMPK 11::GUS* transgenic *A. thaliana* plants were obtained. GUS staining results showed that with no treatment, the GUS signal was barely detectable in the leaf and root (**Figures 4A** and **D**). However, after the plants were treated with GA_3_ for 3 and 12 h, GUS activity increased in the leaves and roots over time (**Figures 4B–E** and F). These data suggested that *GhMPK11* is expressed in response to GA and may be involved in the GA signaling pathway.

### GA_3_ Influences the Chlorophyll Content of *GhMPK11*-Overexpressing Plants

**FIGURE 5:**
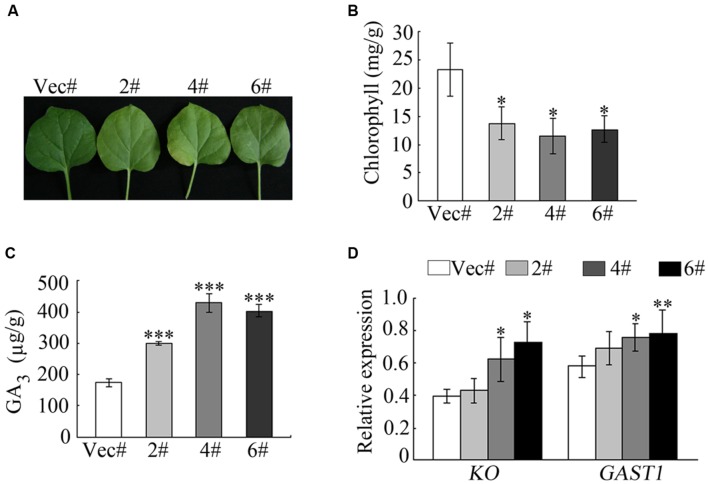
**Overexpression of *GhMPK11* influences the chlorophyll content.** **(A)** Detached leaves from 8-week-old OE and control plants. **(B)** The chlorophyll contents of leaves detached from OE and control plants. **(C)** The GA_3_ contents in OE and control plant leaves. **(D)** GA-related genes expression was detected by qRT-PCR. The data are the means ± SD from three independent experiments. The asterisks indicate statistically significant differences between the transgenic and control plants (^∗^*P* < 0.05; ^∗∗^*P* < 0.01; ^∗∗∗^*P* < 0.001, Student’s *t*-test).

To further analyze the function of *GhMPK11*, transgenic *GhMPK11*-OE *N. benthamiana* plants and transgenic vector control *N. benthamiana* plants were produced. During this process, we observed an interesting phenomenon. When compared with control plants, the leaves of *GhMPK11*-overexpressing plants (OE plants) were less green (**Figure 5A**). In accordance with this phenomenon, the chlorophyll content of control plants was higher than that of OE plants (**Figure 5B**). Considering the sharp increase in *GhMPK11* transcription under GA_3_ treatment and the negative effect of GA on chlorophyll synthesis (Nir et al., 2014), we analyzed the GA_3_ content in leaves from control and OE plants. Interestingly, OE plant leaves showed a higher level of GA_3_ than control plant leaves (**Figure 5C**). To determine whether *GhMPK11* could regulate GA-related genes, the expression levels of *GAST1* and *KO* genes were detected. The results revealed that the control plants exhibited lower expression levels of *GAST1* and *KO* compared to OE plants (**Figure 5D**), which suggested that *GhMPK11* regulates GA-related genes.

### *GhMPK11*-Overexpressing Plants Show Increased Susceptibility to *R. solanacearum* and *R. solani*

**FIGURE 6:**
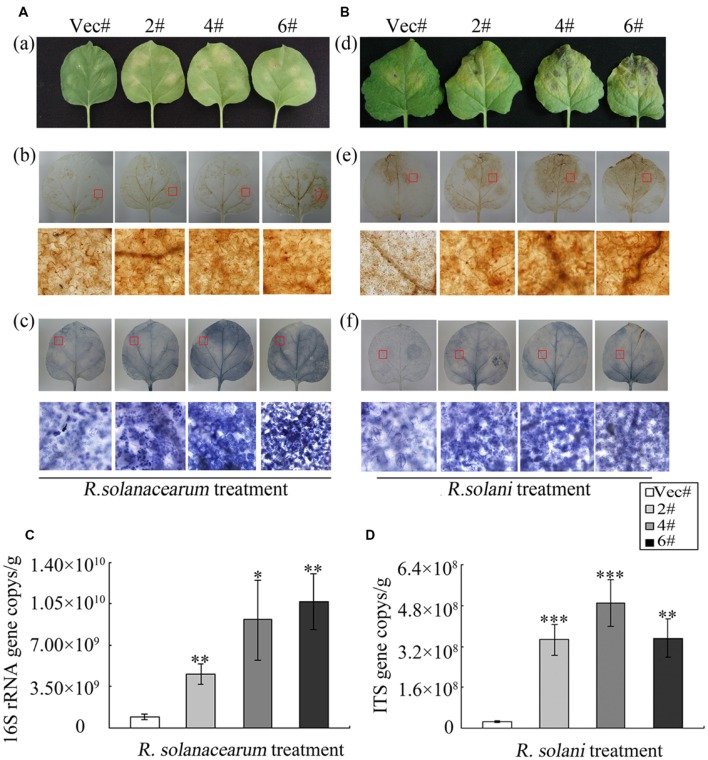
***GhMPK11* Overexpression influences the diseases responses of plants.** **(A)** Leaves infiltrated with *R. solanacearum* and *R. solani* **(B)**. The levels of H_2_O_2_ and 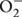 in leaves were determined by DAB (a) and NBT (b) staining, respectively. **(C,D)** Pathogen accumulation after 6 days cultivation determined by qRT-PCR. Data are the mean ± SD from three independent experiments. The asterisks indicate statistically significant differences between the transgenic and control plants (^∗^*P* < 0.05; ^∗∗^*P* < 0.01; ^∗∗∗^*P* < 0.001, Student’s *t*-test).

To explore the role of *GhMPK11* in pathogen resistance in the transgenic tobacco plants, detached leaves from OE and control plants were infiltrated with *R. solanacearum* (OD_600_ = 0.6–0.8) and *R. solani* (10^5^ spores/mL) resuspensions (**Figures 6A,B**). After 6 days of pathogen inoculation, OE plants showed obvious signs of chlorotic and necrotic lesions, whereas control plants did not. A burst of ROS is a common feature of defense responses (Apel and Hirt, 2004; Jones and Dangl, 2006); H_2_O_2_ and superoxide anion (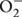) are two types of ROS that can be detected by 3, 3-diaminobenzidine (DAB) and NBT staining, respectively (Thordal-Christensen et al., 1997; Fryer et al., 2002, 2003; Ren et al., 2002). Thus, staining and microscopy analyses were also performed to explain this phenomenon. The DAB and NBT staining results revealed greater staining of the OE plant leaves than control plant leaves after pathogen infiltration (**Figures 6A,B**). Pathogen growth assays performed by qRT-PCR revealed the accumulations of *R. solanacearum* and *R. solani* particles in OE plants were higher than that in control plants after 6 days pathogen inoculation (**Figures 6C,D**). These results indicated that *GhMPK11* overexpression enhanced the susceptibility of transgenic plants to pathogens and ROS.

### The Increased Susceptibility of Plants to Pathogen Treatment is Related to GA_3_ Signaling

**FIGURE 7:**
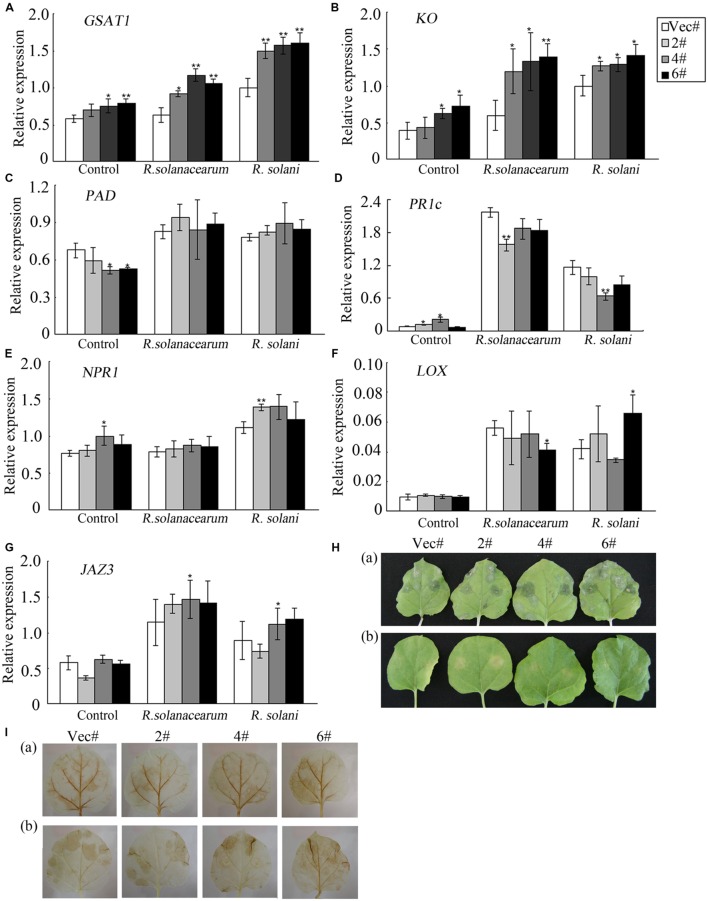
**Expression analysis of hormone-related genes and diseases responses of plants under exogenous GA_3_ treatment.** **(A–G)** The expression levels of the GA-related genes *GAST1* and *KO*; the SA-related genes *PAD*, *PR1c*, and *NPR1*; and the JA-related genes *LOX* and *JAZ3* were detected by qRT-PCR. **(H)** (a) Leaves treated with *R. solani* and 50 mM GA_3_ and (b) leaves treated with *R. solani*. **(I)** DAB staining of leaves shown in **(H)**. The data are the means ± SD from three independent experiments. The asterisks indicate statistically significant differences between the transgenic and control plants (^∗^*P* < 0.05; ^∗∗^*P* < 0.01, Student’s t test).

To evaluate which signaling pathway the *GhMPK11* plants depend on, genes involved in GA, SA, and JA signaling pathways were analyzed by qRT-PCR. Total RNA was extracted from leaves before and after pathogen infiltration. After pathogen infiltration, the expression levels of *GAST1* and *KO*, which are GA-related genes (**Figures 7A,B**), significantly differed, while the expression levels of the SA-related genes *PAD*, *PR1c*, and *NPR1* and of the JA-related genes *LOX* and *JAZ3* only slightly differed (**Figures 7C–G**). These findings suggested that *GhMPK11* might be associated with the GA signaling pathway.

### Exogenous GA_3_ Treatment Increase the Susceptibility of Plants to *R. solani* Treatment

To address the above hypothesis, leaves detached from control and OE plants were infiltrated with *R. solani* resuspension solution and divided into two groups. Group (b) served as a control [**Figure 7H** (b)], and group (a) was also treated with exogenous GA_3_ by placing one piece of cotton soaked in 50 mM exogenous GA_3_ on the petioles [**Figure 7H** (a)]. Four days after infiltration, the leaves in group (a) showed enhanced susceptibility to *R. solani* [**Figure 7H** (a)] compared to the leaves in group (b) [**Figure 7H** (b)]. **Figure 7I** demonstrates increased ROS accumulation in group (a) compared to group (b). Furthermore, control and OE plants showed similar responses. Taken together, these results indicated that exogenous GA_3_ treatment decreased the resistance of plants to *R. solani* treatment and enhanced ROS accumulation.

### *GhMPK11* Overexpression Influences the Transcription of Defense-Related Genes and the Activity of Antioxidant Enzymes

**FIGURE 8:**
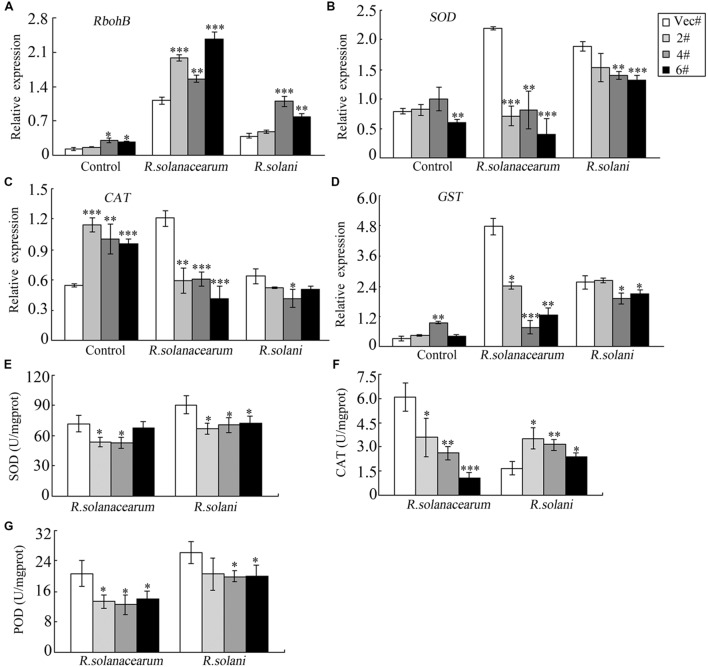
**Relative expression levels and enzymes activities of defense-related genes following diseases infections.** The expression levels of *RbohB* **(A)**, *SOD* **(B)**, *CAT* **(C)** and *GST* **(D)** and the total activities of the antioxidant enzymes SOD **(E)**, CAT **(F)** and POD **(G)** in tobacco plants at 4 days after infiltration with *R. solanacearum* or *R. solani*. The data are the means ± SD from three independent experiments. The asterisks indicate statistically significant differences between the transgenic and control plants (^∗^*P* < 0.05; ^∗∗^*P* < 0.01; ^∗∗∗^*P* < 0.001, Student’s *t*-test).

To further explore the underlying mechanism of pathogen sensitivity, the transcription of the ROS-related gene *RbohB* was analyzed. *RbohB* showed a higher expression level in OE plants after pathogen infiltration (**Figure 8A**). To maintain cellular ROS homeostasis, cells have evolved various enzymes to clear excess ROS, such as SODs, PODs, CAT and GST (Willekens et al., 1997; Apel and Hirt, 2004; Cosio and Dunand, 2009). Here, the expression levels of *NtSOD*, *NtCAT* and *NtGST* were measured by qRT-PCR, and all of these enzymes had higher expression levels in control plants than in OE plants (**Figures 8B–D**) after either *R. solanacearum* or *R. solani* treatment. Similarly, after *R. solanacearum* or *R. solani* treatment, control plants showed higher activity levels of the antioxidant enzymes SOD and POD (**Figures 8E,G**). CAT activity was lower in OE plants compared to control plants when treated with *R. solanacearum*, while CAT activity was higher in OE plants after *R. solani* treatment (**Figure 8F**). All these data indicated that *GhMPK11* overexpression might enhance ROS accumulation by regulating ROS-related genes and antioxidant enzymes under pathogen infiltration.

### *GhMPK11* Overexpression Reduces the Resistance of Transgenic Plants to Oxidative Stress

**FIGURE 9:**
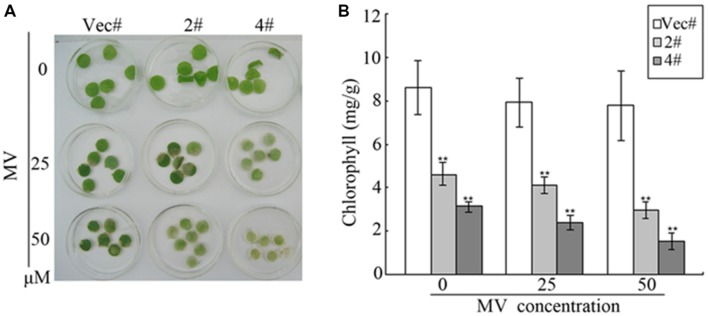
**Oxidative analysis of *GhMPK11*.** **(A)** Leaf disks from control and OE plants soaked in MV solution for 72 h. **(B)** Relative chlorophyll content after MV treatment. The data are the means ± SD from three independent experiments. The asterisks indicate statistically significant differences between the transgenic and control plants (^∗∗^*P* < 0.01, Student’s *t*-test).

Methyl viologen is an herbicide that can cause chlorophyll degradation and cell membrane leakage through ROS production (Kurepa et al., 1998). In our study, MV was used to treat transgenic plants in order to detect the responses of *GhMPK11* to oxidative stress. At the vegetable stage (8-week-old seedlings), after detached leaves were soaked in MV solution for 72 h, leaves from transgenic plants displayed more serious bleaching or chlorosis than those from control plants (**Figure 9A**). Furthermore, **Figure 9B** shows the decreased chlorophyll content of transgenic leaves, further validating the difference in oxidative damage between the transgenic and control plants. These results suggested that *GhMPK11* has a negative influence on oxidative stress responses.

## Discussion

Although many studies have revealed the biological function of specific MAPK proteins in plant defense responses, these studies have mainly focused on MPK3, MPK6 and MPK4, and studies of other MAPK members, especially MAPKs in cotton, are limited. In this study, a group B MAPK gene, *GhMPK11*, was isolated and characterized in cotton. *GhMPK11* transgenic plants showed decreased resistance to the pathogens *R. solanacearum* and *R. solani*; this decreased resistance was verified by the larger necrotic lesions and enhanced pathogen growth observed in infiltrated transgenic plants compared to infiltrate control plants.

Multiple alignments and phylogenetic analyses based on MAPK proteins revealed that GhMPK11 and other MAPK members have a similar protein structure; these analyses confirmed that *GhMPK11* encodes a MAPK. Glory and Murphy (2007) demonstrated that subcellular localization might indicate how a gene interacts with transcription factors to function, offer valuable clues to uncovering its functions, and help understand the complicated pathways that regulate biological processes at the cellular level. *GhMPK11* localized to the nucleus, indicating that it might function with transcription factors. In addition, the observed expression pattern of *GhMPK11* in response to GA_3_, as well as the results of promoter analysis and GUS activity analysis, indicated that *GhMPK11* might be involved in the GA signaling pathway (**Figures 1**–**4**).

To deeply investigate the biological function of *GhMPK11*, *35s::GhMPK11* transgenic plants and 35 s vector control plants were obtained. During the process of transgenic plants growth, an interesting leaf color phenotype was observed. GA is known to have a negative influence on chlorophyll levels in tomato plants (Nir et al., 2014), and compared with wild-type (WT) plants, GA-deficient mutants have darker green leaves (Koornneef and van der Veen, 1980; Koornneef et al., 1985; Peng and Harberd, 1997). Furthermore, GA-deficient plants have higher levels of bioactive GAs than do WT plants (Talon et al., 1990; Peng et al., 1999). Considering these previous studies and our experimental results, we hypothesized that light green leaves were probably caused by the altered GA content in these plants. The higher GA_3_ content and GA-related gene (*KO*, *GAST1*) expression levels observed in OE plants supported this hypothesis (**Figure 5**). The transcriptional suppression of *KO* has been shown to be responsible for decreased GA content (Fukazawa et al., 2000). *GAST1* is a GA-induced gene, and a higher transcript level of this gene correlates with higher GA_3_ content (Shi and Olszewski, 1998). These findings suggested that *GhMPK11* could alter GA_3_ content by regulating GA-related genes.

Except enhanced expression of *GhMPK11* in response to GA_3_, strong induction of *GhMPK11* expression by pathogens was also detected. This expression pattern is consistent with the expression pattern of *AtMPK11* in response to PAMPs (Eschen-Lippold et al., 2012), which suggests that *GhMPK11* might function in defense responses. **Figure 6** showed that *GhMPK11* overexpression could reduce pathogen resistance and enhance pathogen growth. The enhanced susceptibility of *GhMPK11* transgenic plants to *R. solanacearum* was consistent with a previous study of *MPK4* that demonstrated that an *mpk4* mutant exhibited enhanced resistance to *Pseudomonas syringae pv. tomato DC3000*, a Gram-negative plant pathogenic bacterium (Petersen et al., 2000). Although SA and JA are well-established phytohormones that are involved in disease responses (Yang D.L. et al., 2008), emerging evidence has indicated a relationship between GAs and pathogen infection (Qin et al., 2012). Researchers have found that GAs are actively involved in plant immunity and development (Bari and Jones, 2009; Grant and Jones, 2009); however, the underlying mechanism has been only partially elucidated. To determine which pathway was associated with the enhanced susceptibility of transgenic plants, the expression levels of genes related to SA, JA and GA signaling were detected. Significant changes in the expression levels of GA-related genes and slight changes in the expression levels of SA- or JA-related genes indicate that the enhanced susceptibility of transgenic plants to diseases might correlate with the GA signaling pathway. To confirm this result, plants were treated with exogenous GA_3_ while being simultaneously infiltrated with *R. solani*. OE and control leaves displayed enhanced susceptibility and the same phenotype upon *R. solani* infiltration (**Figure 7**). Taken together, these results suggest that exogenous GA_3_ can reduce the influence of endogenous GA_3_ in transgenic plants on disease responses and that *GhMPK11* overexpression can enhance plant susceptibility to pathogen infiltration through the GA signaling pathway. These findings are consistent with the results of a study that found that GA-overproducing *eui* rice appears more susceptible to bacterial and fungal pathogens in the field (Yang D.L. et al., 2008).

A burst of ROS is a common feature of defense responses (Apel and Hirt, 2004; Jones and Dangl, 2006), and plants have developed intricate pathways to fight various environmental stresses by producing more ROS (Coupe et al., 2006). Furthermore, ROS accumulation has been shown to have a negative effect on resistance to necrotrophic pathogens (Yoshioka et al., 2009). In this study, *R. solanacearum* and *R. solani*, which are both necrotrophic pathogens, were used to treat plants (Geraats et al., 2003; Van Loon et al., 2006; Molla et al., 2013; Byth-Illing and Bornman, 2014). Therefore, after pathogen treatment, the accumulation of H_2_O_2_ and 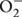 increased greatly (**Figure 6**). To further determine the mechanism of ROS accumulation, ROS-related genes were detected. A previous study showed that down-regulation of *PvRbohB* can decrease 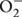 and H_2_O_2_ production in *P. vulgaris* roots (Montiel et al., 2012); conversely, increased expression levels of *RbohB* will lead to more ROS. In addition, the decreased expression level and activity of ROS detoxification enzymes also contributed to ROS accumulation (**Figure 8**). The levels of ROS detoxification enzymes can be regulated by the SCF^SLY 1/GID2^ complex through the GA signaling pathway (Grant and Jones, 2009). Thus, the decreased tolerance of transgenic plants to MV treatment may be associated with elevated ROS levels (**Figure 9**).

Considering these findings, we conclude that the enhanced susceptibility of transgenic plants to pathogen infiltration is a result of enhanced ROS accumulation, which is regulated by *GhMPK11* through the GA signaling pathway. However, although the influence of *GhMPK11* on pathogen resistance was studied here, the comprehensive regulatory mechanism of the defense responses to pathogen infiltration in cotton requires further investigation.

## Author Contributions

XG designed the experiments. FW performed the experiments and analyzed the results with contributions from CW, YY, and HJ. All authors read and approved the final manuscript.

## Conflict of Interest Statement

The authors declare that the research was conducted in the absence of any commercial or financial relationships that could be construed as a potential conflict of interest.

## Supplementary Material

**FIGURE S1 | The subcellular localization of GhMPK11 protein in onion epidermal cells.** Transiently expression of the *35S-GhMPK11-GFP* and *35S-GFP* constructs in onion epidermal cells. Green fluorescence was observed using a confocal microscope. Onion cell nuclei were marked by DAPI staining. Bar = 200 mm.

**TABLE S1 | Primers used in this study.**

## Abbreviations

CaMV: cauliflower mosaic virus
CAT: catalase
DAB: 3,30-diaminobenzidine
ET: ethylene
GA_3_: gibberellin 3
GAST1: GA-stimulated transcript 1
GFP: green fluorescent protein
GST: glutathione S-transferase
GUS: β-glucuronidase
JA: jasmonic acid
KO: ent-kaurene oxidase
MS: Murashige and Skoog
MV: methyl viologen
OE: overexpressing
ORF: open reading frame
qPCR: quantitative real-time PCR
NBT: nitroblue tetrazolium
POD: peroxidase
RbohB: respiratory burst oxidase homolog
ROS: reactive oxygen species
SA: salicylic acid
SOD: superoxide dismutase
WT: wild-type
