# Supplementary figures and images for: Overexpression of Cotton GhMPK11 Decreases Disease Resistance through the Gibberellin Signaling Pathway in Transgenic Nicotiana benthamiana
Source: Front Plant Sci. 2016 May 23;7:689. doi: 10.3389/fpls.2016.00689 (PMC4876126; doi:10.3389/fpls.2016.00689)

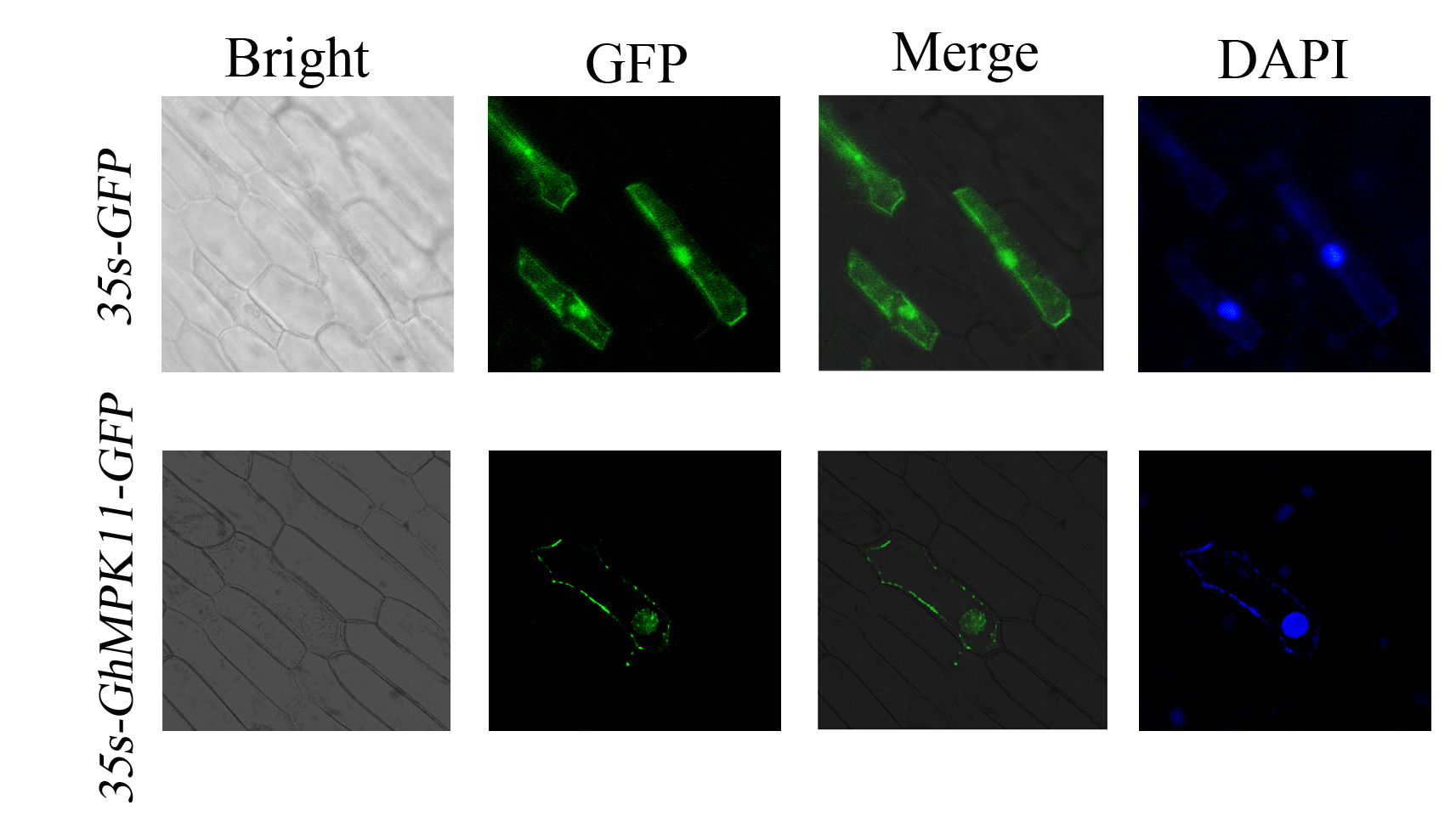

Supplement: Supplementary file 1 [file Image_1.TIF]
