# Supplementary material for: Overexpression of Cotton GhMPK11 Decreases Disease Resistance through the Gibberellin Signaling Pathway in Transgenic Nicotiana benthamiana
Source: Front Plant Sci. 2016 May 23;7:689. doi: 10.3389/fpls.2016.00689 (PMC4876126; doi:10.3389/fpls.2016.00689)
Supplement: Supplementary file 2 [file Table_1.DOC]

| MF | GGTAACCACTTCGAAGTTTCCAG | middle forward |
| --- | --- | --- |
| MR | CTGTGATAAGCCTCAACTGATGC | middle reverse |
| 5N | GCCGACGGGGCGAATAGGAGGGACG | 5'RACE inside |
| 5W | GTTCTCCCTCAGCGGAGGCCG | 5'RACE outside |
| 3N | GCATACTTGGTGAAATGATGACCAG | 3'RACE inside |
| 3W | GGGCTTGCAAGGACGACATCCG | 3'RACE outside |
| N1F | GTGCACTGTAATCAAACTTGAGTATG | intron 1 forward |
| N1R | GCATACAGGCTCCTCATTGATGTC | intron 1 reverse |
| N2F | GTGAGGCTAAGAATTGATTTCC | intron 2 forward |
| N2R | CCAAGTATGCAACCTACCGACC | intron 2 reverse |
| qRB0 | GTCCGGGTAACATACTCAAGTT | promoter RB0 |
| qRB1 | CCTCCTATTCGCCCCGTCGGCCGTGGTGC | promoter RB1 |
| qRB2 | GCAGTAATGACAGGTAAAATCAGAGAGG | promoter RB2 |
| QU | CGGCATGTCAATGGAGTCAACCGG | full length up |
| QD | TCAATCCAGACCCAATTCATTGA | full length down |
| GFPU | GGATCCATGTCAATGGAGTCAACCGG | GFP up |
| GFPD | CTCGAGATGAATTGGGTCTGGATTGT | GFP down |
| RT-up | CGGCATGTCAATGGAGTCAACCGG | RT up |
| RT-down | CCTCCTCACGCGTCTCTGAATTC | RT down |
| 35S-F | CCAAGAAGGTTAAAGATGCAG | forward |
| 35S-R | GAAGACGTGGTTTTAACG | reverse |
| Nb*β*-actin F | TGGACTCTGGTGATGGTGTC | forward |
| Nb*β*-actin R | CCTCCAATCCAAACACTGTA | reverse |
| *GhUBI-*F | TGGACTCTGGTGATGGTGTC | forward |
| *GhUBI-*R | CCTCCAATCCAAACACTGTA | reverse |
| ITS1 | TCCGTAGGTGAACCTGCGG | forward |
| ITS4 | TCCTCCGCTTATTGATATGC | reverse |
| Eub338 | ACTCCTACGGGAGGCAGCAG | forward |
| Eub518 | ATTACCGCGGCTGCTGG | reverse |
